# Facility infrastructure of primary health services regarding tuberculosis control: a countrywide cross-sectional study: Cite this article: Baumgarten A, Hilgert JB, Pinto IC, Zacharias FCM, Bulgarelli AF. (2018) Facility infrastructure of primary health services regarding tuberculosis control: a countrywide cross-sectional study. *Primary Health Care Research & Development* page 1 of 8. doi: 10.1017/S1463423618000646

**DOI:** 10.1017/S1463423618000646

**Published:** 2018-09-17

**Authors:** Alexandre Baumgarten, Juliana B. Hilgert, Ione C. Pinto, Fabiana C. M. Zacharias, Alexandre F. Bulgarelli

**Affiliations:** 1 Faculty of Dentistry, Epidemiology Postgraduate Program, Federal University of Rio Grande do Sul, Porto Alegre, Brazil; 2 School of Nursing at Ribeirão Preto, Public Health Nursing Postgraduate Program at University of São Paulo, São Paulo, Brazil; 3 Faculty of Dentistry, Collective Health Postgraduate Program, Federal University of Rio Grande do Sul, Porto Alegre, Brazil

**Keywords:** health care services, health infrastructure, primary health care, tuberculosis, Unified Health System (SUS)

## Abstract

**Background:**

Tuberculosis (TB) is still a major public health problem in many countries, including Brazil. Primary health care (PHC) services are a set of important services with infrastructure and resources to diagnose, treat, and cure several diseases, including the TB.

**Aim:**

The aim of this study is to analyse aspects of the facility infrastructure of Brazilian PHC, regarding the control and treatment of TB from a countrywide perspective.

**Methods:**

This is a cross-sectional study based on PHC services. Data were collected from 38,812 health centres and were assessed by means of the National Program for Improving Access and Quality Primary Care. The outcome was obtained by the presence and availability of the following infrastructure items: air circulation in the consultation room, refrigerator, individual protective equipment, plastic jar for sputum examination, and TB notification form of the primary care information system. Poisson regression was used to calculate the prevalence ratio.

**Findings:**

Of the 38,812 evaluated centres, only 1628 (4.2%) presented a positive result regarding the outcome. Primary health centres, among all types of centres, presented the highest quality of facility infrastructure for TB control. Centres with large workloads, as well as those that presented a list of offered services and a welcoming consulting room, also presented the highest quality infrastructure. The present study shows that major improvements should be made to the infrastructure to reach a satisfactory TB control in Brazil.

## Introduction

Nowadays, the mortality, incidence, and prevalence rates of tuberculosis (TB) are all decreasing in the eastern Mediterranean and European regions faster than in South America. In South Africa, TB is not fully under control yet (Hermans *et al.,* 2015; Lönnroth *et al.,* 2015). Globally, in the last decade, 58% of pulmonary TB patients were bacteriologically confirmed by means of rapid tests. The rapid test, among others that can diagnose TB, is an important facility infrastructure that usually exists in regions and countries that have been following the recommendations of the World Health Organization (WHO) for TB control. The rapid test was developed in Europe and Brazil (World Health Organization, 2014). Facility infrastructure resources, including structural elements of a health care system that comprises organizational, physical, and technical components, are essential for offering qualified health care (Donabedian, 1980; Scholz *et al.*, 2015). Thus, facility infrastructure is a major component of a health care system to control infectious diseases such as TB.

In Latin America, awareness and investment in TB control health programs that embrace public policies regarding health treatment, disease control, and primary care assistance is part of public health services. Therefore, primary health care (PHC) involves infrastructural and professional resources to carry out the diagnosis and treatment of TB. PHC has good operational indicators regarding the control and management of TB (Bartholomay *et al.*, 2016).

In Brazil, TB has a high rate of incidence with an average of 70,000 new cases per year, that is, an incidence of 62 cases per 100,000 inhabitants. Brazil also has substantial mortality rate (2.3/100 inhabitants), which places Brazil as the 17th country with the highest TB burden in the world (Brasil, 2014). In Brazil, the treatment success rate for new and relapsed cases is 72% and the financing obtained for TB control in 2014 was 68,630 USD$ from the Brazilian National TB program budget (World Health Organization, 2014; Rocha *et al.*, 2015). Although a huge amount of money has been spent, TB still needs to be controlled in Brazil.

The set of services provided by the Brazilian public health system regarding TB control are offered at PHC centres. In Brazil, there are different PHC centres such as primary health centres, family health centres, and advanced health centres. All of them are PHC supporting points that deliver health assistance throughout the national territory. Primary health centres focus on the health of a specific community; family health centres specialize in family health in the counties where they are located; and advanced health centres provide primary and emergency care to the country’s population. All of these options of centres allow the users to have access to TB diagnosis and treatment (Van Hest *et al*., 2014).

Several factors must be observed when a country proposes a national program for TB control. China, for example, more than halved its TB prevalence after scaling up its TB control program. Positive epidemiological results were achieved by means of investments in hospitals and PHC centres using the directly observed therapy (DOT) strategy to ensure better facility infrastructure (Wang *et al.*, 2014). Human resources, physical infrastructure of services, and an appropriate health care model are essential for effective TB control. Based on this combination of factors, DOT and electronic DOT offered by PHC services are needed for TB control. Towards this, surveillance for taking medicines is directly influenced by the presence and activities of professionals in the family health centres and primary health centres (Donabedian 1980; Macaraig *et al.*, 2017). Therefore, assessment of the facility infrastructure for PHC is an important instrument to observe the quality of service provided for users’ care and TB control.

Health services and care management are important matters to be assessed when diseases such as TB need to be controlled. Thus, disease prevention, cure, and control must be followed by the management of pharmacological and infrastructural supplies, utilization of qualified personnel, and coordination of care. Therefore, the assessment surveys presented in this study must be constantly updated countrywide to maintain the procedural assessment of TB control. Furthermore, a nationwide public health system, such as the Brazilian Unified Health System (the Portuguese abbreviation is SUS), ensures constant assessment of its infrastructure to highlight strengths and weaknesses of the system for TB control. In Brazil, PHC services have all the context for TB control, and they can be assessed by using Donabedian’s framework regarding structures, processes, and outcomes of health care. In this framework, infrastructure is the context and refers to the set of facilities where services are delivered to the system user. It includes all types of health centre buildings and physical facilities, amenities, equipment, and personnel (human resources). Improvements in the quality of this construct provide the patient outcome (Donabedian, 1980; Scatena *et al*., 2015). The infrastructure is used to describe the structural elements of the health system, and it consists of one component of the building block of health service delivery (Silva-Sobrinho *et al.*, 2014).

Based on this background, the research question of this study involves assessment of the infrastructure of Brazilian PHC centres for TB control. Thereby, the present study aims to analyse aspects of the facility infrastructure of the Brazilian public health system for the control and treatment for TB from a countrywide perspective.

## Methods

This is a cross-sectional study developed using data from the Brazilian national program for improving access and quality of primary care. The Portuguese abbreviation is PMAQ (Brasil, 2011a). Multiple universities and research institutions have conducted this national program and provided technical resources and personnel in the form of, for example, researchers, evaluators, and tablet computers, for data collection under the supervision of the Brazilian Ministry of Health. The data were collected between May and December 2013 and comprised the first national census of PHC services in Brazil. It comprised 3965 Brazilian municipalities (71.3% of all Brazilian municipalities), and 38,812 PHC centres were studied across these municipalities. This means that of the total 44,754 PHC services throughout all Brazilian municipalities, only 13.3% were not included in this census. This was because these centres refused to participate. The highest number of PHC centres was present in the most populous macro-regions of Brazil: the Northeast (*n*=14,638) and Southeast (*n*=11,943).

For data collection, evaluators were selected and trained uniformly according to a field manual prepared by the Primary Care Department of the Ministry of Health and by the universities and research institutions that carried out the PMAQ. The questionnaire used by the evaluators was specially developed into an Android-based application, which was installed into tablet computers used for data input. The collected data were sent online to a server located in the Ministry of Health in Brasilia (Brazilian capital) and then reviewed to ensure their quality.

The variables included in this study revealed information related to facility infrastructure, equipment, materials, and supplies in PHC services and assistance in Brazil. The maximum quality in the infrastructure for TB control provided the studied outcome.

The variables have been identified based on the subjects of the following questions: 1=‘Are nursing consultations available during the hours and days of unit operation?’; 2=‘Is vaccination available during the hours and days of unit operation?’; 3=‘Is there air circulation in the waiting room?’; 4=‘Do the offices allow privacy to the patient?’; 5=‘Does this health centre have an exclusive refrigerator for vaccines?’; 6=‘Does this health centre have gloves, glasses, face masks, aprons, and caps available?’; 7=‘Does this health centre has plastic jars for sputum examination?’; 8=‘Does this health centre have a health information system for TB?’; and 9=‘Does the health centre have Bacillus Calmette-Guérin (BCG) vaccine available?’. All these nine questions were dichotomous with yes/no response options.

The exploratory variables regarding the health care centre were as follows: (i) type of PHC centre (primary health centre, family health centre, or advanced health centre); (ii) weekdays of attendance; (iii) shifts per day of attendance; (iv) presence of an offered services list at the health centre; (v) indoor signalling for user guidance inside the health centre; and (vi) presence of a welcoming consulting room.

To perform the analysis, Poisson regression with robust variance was used to obtain the adjusted and unadjusted prevalence ratios with their respective 95% confidence intervals. Data were also described by frequencies according to Brazil’s macro-regions (North, Northeast, Midwest, Southeast, and South). All analyses were assessed using the statistical package SPSS v21 (SPSS Inc., Chicago, IL, USA).

This study was submitted to the Federal University of Rio Grande do Sul Ethics Committee and was approved as being ethically and methodologically appropriate (protocol n. 21904/12). All procedures performed in this study were in accordance with the ethical standards of the institutional, national research committee, and with the 1964 Helsinki declaration and its later amendments or comparable ethical standards.

## Results

In the present study, 38,812 (86.7%) PHC centres of the SUS in Brazil were assessed. This study can be characterized as a census of the Brazilian public PHC services and of the infrastructure for TB control. Brazil is divided into five macro-regions with different characteristics. The North and Northeast are larger regions with higher number of PHC centres widespread in their territories.

However, these regions also have more social vulnerability and presence of physical barriers to access health centres such as the need for long distance travel by boat. The South, Southeast, and Midwest regions have higher population density and less number of PHC centres. They have less population vulnerability and average indicators for TB morbidity. From the social and economic perspectives, the gross national product (GNP) and human development index (HDI) are better in the South and Southeast regions.

Variables used to set the maximum quality of the infrastructure for TB control are presented in Table 1. The northernmost regions depict the worst results in seven of the nine assessed items. The items were ‘no nursing consultation’ (22.2%), ‘available vaccination’ (37.7%), ‘air circulation’ (49.9%), ‘privacy for consultation’ (29.0%), ‘refrigerator available’ (44.6%), ‘individual protective equipment’ (44.1%), and ‘plastic jar for sputum examination’ (59.6%). The Northeast region showed the worst results in one of the nine items assessed, ‘presence of the tuberculosis notification form’ (46.6%), and the Southeast was the worst in one item, ‘BCG-ID vaccine available’ (55.8%).

**Table 1:** Items assessed to set the maximum quality in the infrastructure for tuberculosis control. Stratified by geographical macro-region. Brazil, 2017

Variable		Midwest *n* (%)	Northeast *n* (%)	North *n* (%)	Southeast *n* (%)	South *n* (%)	Brazil *n* (%)
Available nursing consultation	No	217 (8.0)	1430 (9.8)	713 (22.2)	1112 (9.3)	647 (10.2)	4119 (11.9)
	Yes	2489 (92.0)	13,208 (90.2)	2497 (77.8)	10,830 (90.7)	5668 (89.8)	34,692 (88.1)
Available vaccination	No	625 (23.1)	2521 (17.2)	1211 (37.7)	3405 (28.5)	1666 (26.4)	9428 (26.6)
	Yes	2081 (76.9)	12,117 (82.8)	1999 (62.3)	8537 (71.5)	4649 (73.6)	29,383 (3.4)
Air circulation	No	1117 (41.3)	5974 (40.8)	1601 (49.9)	3367 (28.2)	1615 (25.6)	13,674 (37.1)
	Yes	1589 (58.7)	8664 (59.2)	1609 (50.1)	8576 (71.8)	4700 (74.4)	25,138 (62.9)
Privacy to consultation	No	456 (16.9)	2788 (19.0)	930 (29.0)	1727 (14.5)	917 (14.5)	6818 (18.8)
	Yes	2250 (83.1)	11,850 (81.0)	2280 (71.0)	10,216 (85.5)	5398 (85.5)	31,994 (81.2)
Refrigerator available	None	783 (29.1)	5194 (35.6)	1417 (44.6)	3661 (30.9)	1893 (30.1)	12,948 (34.1)
	One or more	1907 (70.9)	9386 (64.4)	1763 (55.4)	8178 (69.1)	4404 (69.9)	25,638 (65.9)
Individual protective equipment	Never or sometimes	1016 (37.5)	6188 (42.3)	1415 (44.1)	3450 (28.9)	1741 (27.6)	13,810 (36.1)
	Always	1690 (62.5)	8450 (57.7)	1795 (55.9)	8492 (71.1)	4574 (72.4)	25,001 (63.9)
Plastic jar for sputum examination	Never or sometimes	966 (35.7)	8138 (55.6)	1914 (59.6)	3166 (26.5)	2392 (37.9)	16,576 (43.1)
	Always	1740 (64.3)	6500 (44.4)	1296 (40.4)	8776 (73.5)	3923 (62.1)	22,235 (56.9)
Tuberculosis notification form	Never or sometimes	1113 (41.1)	6819 (46.6)	1441 (44.9)	4743 (39.7)	2872 (45.5)	16,998 (43.6)
	Always	1593 (58.9)	7819 (53.4)	1769 (55.1)	7199 (60.3)	3443 (54.5)	21,823 (56.4)
BCG-ID vaccine available	Never or sometimes	1129 (41.7)	7854 (53.7)	1738 (54.1)	6665 (55.8)	3495 (55.3)	20,881 (52.1)
	Always	1577 (58.3)	6782 (46.3)	1472 (45.9)	5275 (44.2)	2820 (44.7)	17,926 (47.9)

Due to some losses, not all covariates add up the total *N* which is 38,812.

The prevalence of the outcome in Brazil was 4.2%. This means that, among the 38,812 PHC centres evaluated, only 1628 (4.2%) presented a positive result in the nine items assessed, which composed the outcome. Based on macro-regions, the results are as follows: Midwest=186 (0.5%); Northeast=611 (1.6%); North=160 (0.4%); Southeast=460 (1.2%); and South=211 (0.6%).

In the crude analysis, all the explanatory variables were associated with the highest quality in the infrastructure for TB. After adjustment, only indoor signalling for user guidance was not associated. When compared with the family health centre, the results of the adjusted analysis showed higher prevalence of establishments of the primary health centre type having the highest quality infrastructure for TB control, which is different from the advanced health centre. With regard to working time, covering the weekdays and shifts, the outcome was better for establishments with larger workloads, as well as for those that presented a list of offered services and a welcoming consulting room (Table 2).

**Table 2:** Crude and adjusted PR for quality in TB infrastructure and associated factors. Brazil, 2017

		Crude PR		Adjusted PR	
Variables		PR	95% CI	PR	95% CI
Type of primary health care centres	Family health centre	1		1	
	Primary health centre	1.85	1.63–2.09	1.53	1.36–1.76
	Advanced health centre	0.27	0.13–0.58	0.34	0.16–0.73
	Other	1.14	0.89–1.44	1.15	0.90–1.46
Weekdays of attendance	1–4 days	1		1	
	5 days	7.40	4.20–13.0	4.13	2.31–7.38
	6–7 days	6.27	3.46–11.3	3.80	2.08–6.97
Shifts of work	1 Shift	1		1	
	2 Shifts	2.96	2.32–3.78	1.87	1.44–2.42
	3 Shifts	3.34	2.45–4.56	2.04	1.48–2.82
List of offered services	No	1		1	
	Yes	1.69	1.53–1.86	1.46	1.32–1.62
Indoor signalling for user guidance	No	1		1	
	Yes	1.29	1.17–1.42	1.01	0.91–1.12
Welcoming consulting room	No	1		1	
	Yes	1.44	1.30–1.59	1.23	1.11–1.37

(PR=prevalence ratio; CI=confidence interval)

Upon evaluating the type of unit, the primary health centre was associated with the highest quality in infrastructure for TB control in all macro-regions, except for the Southeast. Moreover, the variables of weekdays and shifts of attendance were associated with higher working time in the Northeast and Southeast. The list of offered services was associated with the Northeast, North, and Southeast regions. Regarding the variable of indoor services, signalling was only associated with the Northeast region, and the variable of the welcoming consulting room was not associated with the Midwest region. In Brazil, occurrence of the minimum and maximum prevalence of infrastructure items for TB control is similar in the macro-regions. Maximum prevalence of the available nursing consultation was in the Midwestern (97.9%) and Southern (96.6%) regions. The minimum prevalence of individual protective equipment (12.7%) and BCG-ID vaccine available (18.5%) occurred in the North (Tables 3 and 4).

**Table 3:** Crude and adjusted PR for quality in TB infrastructure and associated factors by macro-region. Brazil, 2017

	Midwest		Northeast		North		Southeast		South	
	PR	95%CI	PR	95%CI	PR	95%CI	PR	95%CI	PR	95%CI
Type of primary health care centres										
Family health centre	1		1		1		1		1	
Primary health centre	1.73	1.12–2.68	1.56	1.29–1.90	2.46	1.59–3.81	1.57	1.19–2.08	1.01	0.72–1.42
Advanced health centre	0.33	0.04–2.32	0.73	0.47–1.13	2.39^a^	1.26–4.52	0.81	0.29–2.22	0.14	0.02–1.07
Other	1.12	0.60–2.09	0.86	0.54–1.35	–	–	1.46	0.91–2.32	0.58	0.27–1.22
Weekdays of attendance										
1–4 days	1		1		1		1		1	
5 days	5.79	0.76–44.0	3.05	1.50–6.23	1.98	0.29–13.3	3.90	0.95–15.8	1^b^	
6–7 days	3.49	0.43–28.1	2.64	1.22–5.71	1.48	0.20–10.8	4.32	1.02–18.3	0.96	0.45–2.05
Shifts of attendance										
1 Shift	1		1		1		1		1	
2 Shifts	0.57	0.34–0.95	3.01	1.89–4.80	1.38	0.83–2.31	2.10	1.11–3.99	2.88	1.06–7.85
3 Shifts	0.66	0.27–1.61	4.55	2.51–8.24	1.15	0.50–2.67	2.27	2.12–4.62	3.08	0.99–9.60
List of offered services										
No	1		1		1		1		1	
Yes	1.15	0.85–1.56	1.52	1.28–1.80	1.60	1.12–2.27	1.79	1.48–2.17	0.97	0.74–1.28
Indoor signalling for user guidance										
No	1		1		1		1		1	
Yes	1.18	0.87–1.59	1.35	1.15–1.59	0.87	0.61–1.24	0.79	0.65–0.95	0.98	0.74–1.28
Welcoming consulting room										
No	1		1		1		1		1	
Yes	0.76	0.54–1.08	1.46	1.18–1.81	1.57	1.11–2.21	1.53	1.27–1.84	1.58	1.19–2.09

a Specifically for North region, it was united the categories advanced health centre and other.

b Specifically for South region, it was categorized in 1–5 days and 6–7 days.

(PR=prevalence ratio; TB=tuberculosis)

**Table 4:** Occurrence of minimum/maximum prevalence of tuberculosis infrastructure items available within geographical macro-region. Brazil, 2017

Macro-region^a^	Nursing consultation (%)	Availability vaccination (%)	Air circulation (%)	Privacy (%)	Refrigerator available (%)	Individual protective equipment (%)	Plastic jar (%)	Tuberculosis notification form (%)	BCG-ID vaccine available (%)
North									
Minimum	51.9	46.8	35.1	46.8	31.8	12.7	19.6	19.0	18.5
Maximum	96.2	90.0	69.4	90.0	83.8	77.0	73.2	83.2	80.4
Northeast									
Minimum	76.6	65.4	42.7	64.5	31.3	35.5	15.4	29.4	20.6
Maximum	96.6	97.0	68.9	91.9	93.2	71.3	64.4	75.6	80.8
Midwest									
Minimum	86.2	70.3	45.2	64.8	55.4	44.8	49.6	50.1	51.4
Maximum	97.9	79.8	65.9	86.7	71.8	65.3	74.9	74.5	64.1
Southeast									
Minimum	78.1	53.2	60.3	77.8	51.6	52.4	66.0	54.0	19.7
Maximum	96.3	79.0	74.3	90.6	78.6	83.2	84.3	67.4	58.8
South									
Minimum	85.4	71.8	72.7	80.3	67.3	66.6	55.7	39.1	32.4
Maximum	95.0	76.8	78.6	91.5	74.3	77.5	70.9	64.4	53.7

a According to the methodology mentioned

## Discussion

This is the first nationwide study that describes and assesses the infrastructure for TB control in Brazil. Until the present, no other study of such magnitude and national scope has sought to assess PHC centres’ services and infrastructure for TB control nationwide. A census of the Brazilian public PHC infrastructure was performed in 38,812 PHC centres. The results of the present cross-sectional study are based on data from the PMAQ (Brasil, 2011a), indicating that different perspectives of TB control can be influenced by the facility infrastructure and services offered to users by the public health service. Some other PMAQ descriptive results were published in Brazilian local reports and statistics, but none of them proposed a composite facility infrastructure outcome regarding TB control to deeply investigate this research subject.

It is important to highlight that based on the data provided by the PMAQ, nine variables were used to set the maximum quality infrastructure for TB control in Brazil (research outcome). Among them, the presence of air circulation in family health centres is higher in the Southeast region, and it was observed that in some macro-regions such as the North and Northeast, <45.2% centres have air circulation in their health services buildings. Air circulation inside buildings is essential to avoid airborne TB transmission (Brasil, 2011b). The results are opposite regarding the presence of the TB notification form and availability of BCG vaccine in the primary health centres. A refrigerator, which is essential for TB diagnosis and control in terms of storing vaccines and sputum tests, was present in only 65.9% centres in Brazil as a whole, which is a weakness of the infrastructure for TB control. The presence of the refrigerator corroborates the adequate infrastructure for TB control (Andrade *et al.*, 2013). According to Donabedian’s framework, laboratory strengthening and investment in diagnosis facilities have potential to improve the infrastructure so that it can provide quality access to disease control (Donabedian, 1980; World Health Organization, 2014).

The results of the present study point out that high quality of TB control is associated with some aspects of the primary health infrastructure of the SUS. These aspects related to high quality, such as a good supply facility system, adequate physical infrastructure, and outreach programs, need to be offered according to the literature and the WHO manual on TB Control (World Health Organization, 2014; Brasil, 2015; Scatena *et al*., 2015; Scholz *et al.*, 2015). The theoretical model constructed in the present study reflects the higher quality of infrastructure as the outcome of satisfactory TB control in the five Brazilian macro-regions. It is important to note that each of these five macro-political regions has different GNP and HDI. Of the total GNP of Brazil in 2014, the South was responsible for 16.2%, Southeast for 55.2%, North for 5.3%, Northeast for 13.5%, and Midwest for 9.8% (Brasil, 2015). This fact can be associated with the differences in infrastructures and services presented in this study. Differences among health service resources associated with TB control are a reality in Brazil (Bartholomay *et al.,* 2016). The differences in social determinants are associated with the maintenance of higher levels of TB in Brazil. Development and enforcement of public health system policies, investment in better physical infrastructure, and identification of barriers to improve access to diagnosis and treatment for TB control are highlighted as part of strategies to control the disease (Lönnroth *et al.*, 2010; Van Hest *et al*., 2014; Scholz *et al.*, 2015; Bartholomay *et al*., 2016). Brazil has been making efforts towards primary health infrastructure to control TB, but the disparities in social determinants nationwide are barriers to ensuring health treatment access. In these situations, provision of low-threshold and accessible services while improving DOT is necessary (Van Hest *et al*., 2014).

Better infrastructure for TB control is associated with health services with extended workloads involving three daily shifts of work. This might be associated with better access to treatment and care when the service user looks for assistance. Better access reflects better TB control (Van Hest *et al*., 2014). SUS users who are taking TB medicine may need to visit the health centre many times a week, and so three shifts of work and higher weekday attendance enhance the access to disease control (Lafaiete *et al*., 2011).

The type of health centre reflects different models of assistance, which are associated with diverse shifts of work and the presence of welcoming consulting. A study comparing different models of assistance showed that gaps exist in the PHC model regarding the diagnosis and control of TB (Andrade *et al.*, 2013). The present study showed that these gaps might indicate the absence of a list of offered services, indoor service signalling, and welcoming consulting room. The Brazilian program for TB control (the abbreviation in Portuguese is PNCT) indicates that satisfactory control of the disease can be reached by means of simultaneous decentralization of actions based on the PHC assumptions and good infrastructure of primary health centres (Brasil, 2008).

In addition, health centres that provide the list of offered services and welcoming actions in a private consulting room are associated with high quality of TB control. However, a welcoming consulting room is not associated with high quality of TB care only in the Midwest region. These facts are concerns that could be associated with infrastructure management and coordination of care, both achieved by means of strong PHC development in addition to decentralization and organization of assistance (Donabedian, 1980; Lönnroth *et al.*, 2015; Silva-Sobrinho *et al.*, 2014; Van Hest *et al*., 2014; Scholz *et al.*, 2015). The required facility infrastructure management, as well as welcomeness and the available list of offered services, can be found in the family health centres (Ponce *et al.*, 2013).

Family health centres have better outcomes regarding TB control. Furthermore, Brazilian primary health centres seem to have the highest quality infrastructure for TB when compared with the infrastructure of advanced health centres. Previous literature shows that TB control faces some barriers that need to be improved when actions are centralized only in advanced health centres (Ben *et al.*, 2013; Ponce *et al.*, 2013; Silva-Sobrinho *et al*., 2014). This is because advanced health centres do not provide the integrality of care required for family care assistance (Silva-Sobrinho *et al.*, 2014). From another perspective, advanced health centres have better performance as regards TB diagnoses, and it needs to be reflected through primary health services for better disease control and continuity of treatment (Ponce *et al*., 2013).

In all Brazilian macro-regions, except for the South, primary health centres have reached the outcome required (highest quality in infrastructure) for TB control. One study showed that in one municipality in the South of Brazil, the PHC infrastructure failed when the issue was human and physical resources for TB control (Silva-Sobrinho *et al.*, 2014). Thus, improvements are necessary for training human resources for TB control in this macro-region (Ben *et al*., 2013; Silva-Sobrinho *et al.*, 2014). This can be achieved by means of a management model that focusses on constant assessment of the health services infrastructure. Constant evaluation of the infrastructure enriches the quality of health services and the control of infectious diseases (Mtui and Spence, 2014). Bulage *et al.* (2014) identified the effective aspects as regards the quality of infrastructure and service for TB control, which are based on overall organizational characteristics. Aspects such as TB notification forms and availability of TB drugs were identified in all Brazilian macro-regions.

Concerning the variables of weekdays of attendance and shifts, the Northeast and Southeast presented high quality of TB control because they reflect higher working time, which provides users better access to treatment. Previous studies showed that some aspects of PHC such as quality of infrastructure promote better TB control (Andrade *et al.*, 2013; Mtui and Spence, 2014; Silva-Sobrinho *et al.*, 2014).

The limitation of the present study is related to the fragmentation of the evaluation process, which was conducted by different universities and, consequently, by different evaluating teams. The strengths of this study meet the main strongholds of a health care services census. This is a nationwide program that is unprecedented, and inductor of processes aimed towards increasing the capacity of the local, state, and federal management, as well as that of PHC, to offer services that ensure greater accessibility for TB treatment to the population. Further, based on these results, the program will transfer funding to municipalities to ensure improved access and quality of provision of the studied services.

## Summary and final considerations

The present study showed that a family health centre with five weekdays in three shifts of attendance with a welcoming consulting room reflects high quality of facility infrastructure and services for TB control. This is because differences in the Brazilian territory regarding the presence of high-quality facility infrastructure are a reality in some Brazilian macro-regions. The results of the present study corroborate the fact that gaps still exist for controlling TB, and much improvement is needed to qualify PHC services in Brazil. This study identifies the weaknesses in Brazil regarding TB control, and it can be an example to guide future improvements even in other countries to develop better facility infrastructure regarding TB control.

## Authors’ Contribution

All authors contributed during every step of the research until the writing of the manuscript. All authors read and approved the final manuscript.

## Financial Support

This study was funded by Brazilian Ministry of Health.

## Conflicts of Interests

None.
